# The impact of sleep deprivation and nighttime light exposure on clock gene expression in humans

**DOI:** 10.3325/cmj.2011.52.594

**Published:** 2011-10

**Authors:** Pavel Kavčič, Bojan Rojc, Leja Dolenc-Grošelj, Bruno Claustrat, Kristina Fujs, Mario Poljak

**Affiliations:** 1 Institute of Clinical Neurophysiology, Division of Neurology, University Medical Centre, Ljubljana, Slovenia; 2 Hormone Laboratory, Nuclear Medicine Centre, Bron, France; 3 INSERM, U846, Stem Cell and Brain Research Institute, Department of Chronobiology, Bron, France; 4 Institute of Microbiology and Immunology, Medical Faculty of Ljubljana, Ljubljana, Slovenia

## Abstract

**Aim:**

To examine the effect of acute sleep deprivation under light conditions on the expression of two key clock genes, *hPer2* and *hBmal1,* in peripheral blood mononuclear cells (PBMC) and on plasma melatonin and cortisol levels.

**Methods:**

Blood samples were drawn from 6 healthy individuals at 4-hour intervals for three consecutive nights, including a night of total sleep deprivation (second night). The study was conducted in April-June 2006 at the University Medical Centre Ljubljana.

**Results:**

We found a significant diurnal variation in *hPer2* and *hBmal1* expression levels under baseline (*P* < 0.001, F = 19.7, df = 30 for *hPer2* and *P* < 0.001, F = 17.6, df = 30 for *hBmal1*) and sleep-deprived conditions (*P* < 0.001, F = 9.2, df = 30 for *hPer2* and *P* < 0.001, F = 13.2, df = 30 for *hBmal1*). Statistical analysis with the single cosinor method revealed circadian variation of *hPer2* under baseline and of *hBmal1* under baseline and sleep-deprived conditions. The peak expression of *hPer2* was at 13:55 ± 1:15 hours under baseline conditions and of *hBmal1* at 16:08 ± 1:18 hours under baseline and at 17:13 ± 1:35 hours under sleep-deprived conditions. Individual cosinor analysis of *hPer2* revealed a loss of circadian rhythm in 3 participants and a phase shift in 2 participants under sleep-deprived conditions. The plasma melatonin and cortisol rhythms confirmed a conventional alignment of the central circadian pacemaker to the habitual sleep/wake schedule.

**Conclusion:**

Our results suggest that 40-hour acute sleep deprivation under light conditions may affect the expression of *hPer2* in PBMCs.

The prevalence of sleep deprivation and nighttime light exposure in the industrialized world appears to be on the rise (1). It is estimated that between 15 and 30% of the workforce in industrialized countries operates outside standard daytime hours (2). Sleep deprivation has significant consequences for public health, safety, and quality of life. Commonly reported problems during periods of sleep deprivation are excessive daytime sleepiness, fatigue, and difficulty in concentrating. Recent epidemiological studies have indicated that workers who experience sleep deprivation, circadian disruption, and exposure to light at night are at increased risk of cardiovascular disease, diabetes, and certain cancers (3-5). Development of cancer in circumstances when circadian rhythms are disrupted is thought to be affected by circadian clock genes (6,7).

Circadian rhythms exist in all mammals, including humans, and are controlled by a biological clock. The center of the biological clock is located in the suprachiasmatic nucleus (SCN) of the anterior hypothalamus and oscillates self-sustained (8). It is synchronized with the 24-hour day by environmental time cues, especially light via the retino-hypothalamic tract, locomotor activity, and meal times (9,10). SCN transmits the timing signal to peripheral tissues via neural and humoral mechanisms and so synchronizes independent self-sustained circadian oscillators that exist in most peripheral tissues (11-14). In this way, peripheral tissues can appropriately respond, according to their specific function, to the correct time of day (15-17). Melatonin and cortisol secretion is regulated in this manner via the SCN-paraventricular nucleus of the hypothalamus axis (18). Positive and negative transcriptional-translational feedback loops of clock genes represent molecular components of the circadian clock system. At least 10 genes essential for mammalian circadian clock function have been identified. The positive regulators in mammals are *Clock* and *Bmal1*, whereas *Per1-3*, *Cry1-2,* and *Dec1-2* are involved in the negative feedback loop (7,14,19). The first human study showed a circadian profile of clock gene expression in oral mucosa and skin, suggesting their functional importance in man (20). Circadian expression of clock genes was also reported in human peripheral blood mononuclear cells (PBMC) (21). Recent studies have suggested that clock gene expression profiles in human PBMCs may be a useful marker for assessing circadian rhythm in humans (22-25).

The light-dark cycle is known to be the primary environmental signal that synchronizes circadian rhythms (8). It was shown that a 6.5-hour long bright light stimulus at 50 lux can already produce a phase shift in melatonin rhythm (26). Little is known about the effect of nighttime light exposure or sleep deprivation on human clock gene expression. In nocturnal rodents, it has been suggested that nighttime dim light exposure or acute sleep deprivation alone can alter gene expression in the SCN (27,28). However, in humans it is impossible to assess clock gene expression in the SCN. A recent human study assessed clock gene expression in PBMCs throughout a normal sleep/wake cycle and during a constant routine protocol (absence of sleep/darkness episodes) and found no major differences in circadian rhythmicity (29). Another human study demonstrated that the circadian pattern of clock gene expression in PBMCs adapted to a shifted sleep/wake schedule. The changes were apparent as of 3 days on the night shift schedule (30). The latter study indicates that the assessment of clock gene expression in human PBMCs might be a good indicator of central adaptation to the shifted sleep/wake schedule.

To our knowledge, the impact of sleep deprivation, together with nighttime light exposure, on human PBMCs has not been studied before. The aim of the study was to evaluate whether 40-hour acute sleep deprivation under light conditions (mimicking the “awake” night) affects the daily expression of clock genes in human PBMCs.

## Methods

### Participants

Seven healthy men, median age 26 years (range 25-35 years), were enrolled in the study as volunteers. They all experienced a normal sleep/wake cycle (28) and completed a sleep diary for three weeks before entering the protocol. Their habitual sleep/wake schedule was approximately between 23:00 and 7:00 hours. They were neither shift workers nor sleep deprived for at least 3 weeks prior to the study. All were healthy non-smokers and abstained from consuming alcohol, caffeine, and other psycho-stimulant beverages during the study protocol. They had not taken any medication for at least 4 weeks prior to the study.

The selection of participants was made on the first, adaptation, night, which also served as the baseline night. One participant was excluded due to an anxiety attack. On the first night, participants slept in the sleep laboratory at the University Medical Centre Ljubljana, and classical polysomnography (PSG) was performed. PSG included electroencephalogram, electro-oculogram, and electromyogram channels. Respiration (nasal airflow, thoracic, and abdominal level), Sao_2_ (oxygen saturation), electromyogram of the anterior tibial muscles, and electrocardiogram recordings were analyzed in order to exclude sleep-related breathing disorders or periodic limb movement during sleep. An apnea-hypopnea index below 5 and periodic leg movement index below 5 were prerequisites for study entry. The study was performed in April-June 2006. All participants gave written consent before participation. The study protocol was approved by the Ethical Committee of the University of Ljubljana Medical School.

### Experimental procedure

The investigation took place over 56 hours; including a baseline night, first day, night and a day of total sleep deprivation (second night and day), and a recovery night (third night). During the first night (baseline night), the participants slept in the sleep laboratory and were recorded with PSG (from 23:00 to 7:00) to exclude any sleep disorders. There was no computer, television, radio, or telephone in the room, and cell phones were not permitted. Following PSG, participants were continuously awake from 7:00 for 40 hours. Light intensity during the daytime period was about 500 lux. All participants followed the same daily schedule during daytime; they were allowed to be moderately physically active and had breakfast, lunch, and dinner at 8:00-9:00, 13:00-14:00, and 19:00-20:00 hours, respectively. Participants underwent a battery of other tests under strict control, with the same timetable for all participants. The night of total sleep deprivation was carried out in our laboratory in groups of two. During the night of sleep deprivation, participants were kept under sedentary (regularly seated) and constant environmental conditions with a maximum light intensity of 50 lux. During the 40-hour deprivation, two physicians continuously supervised participants in order to keep them awake. At the end of the 40-hour period of sleep deprivation, participants were allowed to sleep. Recovery sleep (from 23:00 to 7:00) was monitored with PSG to demonstrate the rebound effects of sleep deprivation.

### Blood samples

An indwelling catheter was placed in the antecubital vein and blood samples were taken at 4-hour intervals for three consecutive nights, from 23:00 on the baseline night until 7:00 on the recovery night. The volume of drawn blood, which was about 150 mL per participant, was replaced by saline. If catheter stopped working it was removed and blood was taken directly from a vein. However, before the recovery night the catheter was placed back in the vein in order not to wake up the participants during blood sampling. Blood samples for melatonin and cortisol were collected in 8 mL-Vacutainer CPT tubes (containing sodium heparin; Becton Dickinson, Franklin Lakes, NJ, USA) and centrifuged for 30 minutes at 1600 × g at room temperature to isolate plasma. Plasma was stored at -20°C until assayed. Blood samples for RNA isolation were collected into PAXgene Blood RNA tubes (Qiagen, Hilden, Germany) and stored at -80°C until RNA isolation.

### RNA isolation and quantitative real-time reverse-transcription polymerase chain reaction (qRT-PCR)

Total cellular RNA was isolated from PBMC samples using the PAXgene Blood RNA Kit (Qiagen, Hilden, Germany) according to the manufacturer's instructions, including a special procedure for thawing frozen PBMCs. During RNA isolation, traces of residual DNA were removed by an additional on-column DNA digestion using the RNase-Free DNase Set (Qiagen). In all samples, RNA concentrations were measured using the RNA Quant-iT RNA Assay Kit (Invitrogen, Carlsbad, CA, USA) on a Qubit fluorometer Q32857 (Invitrogen), according to the manufacturer's instructions.

The levels of expression of two clock genes, *hPer2* and *hBmal1*, as well as the in-house gene *36B4*, were determined using one-step qRT-PCR on a Light Cycler 2.0 Instrument (Roche Applied Science, Mannheim, Germany) using the QuantiTect SYBR Green RT-PCR Kit (Qiagen), which allows closed one-tube reverse transcription and amplification of cDNA using Omniscript and Sensiscript reverse transcriptases. The qRT-PCR was performed using 100 ng of RNA per reaction and 1 μM of the previously described primers (31). Cycling parameters were 20 minutes at 50°C (reverse transcription), followed by 40 cycles at 94°C for 15 seconds, 55°C for 20 seconds, and 72°C for 30 seconds. The specificity of PCR products was confirmed using melting curve analysis. Melting temperatures were 84°C for *36B4*, 83°C for *hPer2,* and 78°C for *hBmal1*. Expression levels were normalized to the levels of the constitutively expressed non-rhythmic control *36B4* gene in humans, as described previously (30). The relative abundance of mRNA (messenger RNA) was calculated using a standard curve method.

Primer sequences were for *hPer2* were 5′-GCAGGTGAAAGCCAATGAAG and 5′-CACCGCAAACATATCGGCAT, for *hBmal1* 5′-AAGGATGGCTGTTCAGCACATGA and 5′-CAAAAATCCATCTGCTGCCCTG, and for 36B4 5′-AATCCCTGACGCACCGCCGTGATG and 5′-TGGGTTGTTTTCCAGGTGCCCTCG.

Melatonin and cortisol were determined using radioimmunoassays, as described previously (32).

### Statistical analysis

Clock gene expression is reported as a relative ratio of the constitutively expressed *36B4* gene (31). Diurnal variations of mRNA expression and cortisol levels were tested using one-way repeated measures analysis of variance (ANOVA). Diurnal variations of melatonin levels were tested using the Friedman test since the variables did not follow the normal distribution. The area under the curve value was calculated for melatonin and cortisol levels on the first and second night and compared by Wilcoxon matched pairs test. The single cosinor method adapted to a 24-hour period was used for analyzing circadian rhythms of *hPer2* and *hBmal1* under baseline and sleep-deprived conditions (33). The rhythm characteristics estimated by this method include the acrophase (timing of the cosine maximum), mesor (mean of the oscillation), and amplitude. Goodness of fit (R^2^) was also obtained. A statistically significant circadian oscillation was considered if the 95% confidence interval for the amplitude did not include the zero value. The results are expressed as means ± standard error.

## Results

### Diurnal variation of melatonin and cortisol in healthy participants

**Figure 1:**
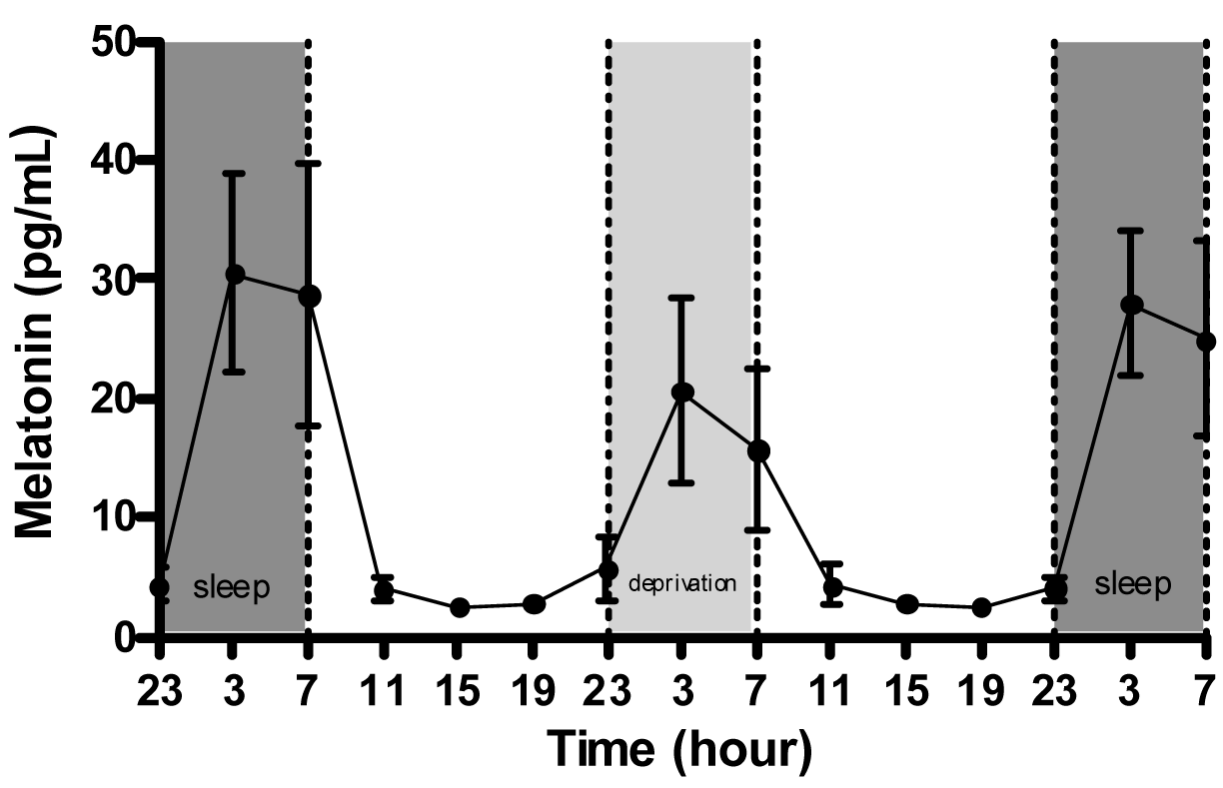
Plasma melatonin levels during the 56-hour sampling period. Each time point represents the mean ± standard error. These profiles showed significant daily variation (*P* < 0.001). The calculated area under the curve was significantly smaller during the second night in comparison to the first night (*P* = 0.03).

**Figure 2:**
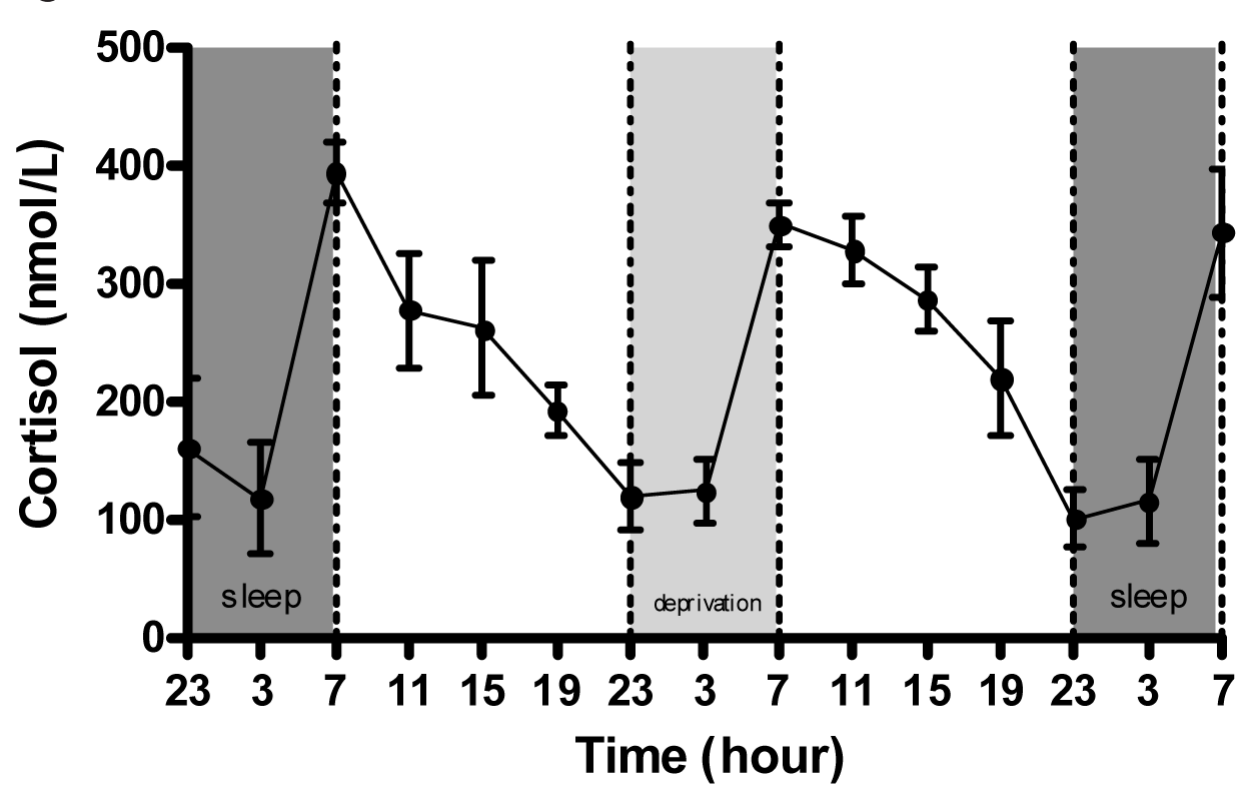
Plasma cortisol levels during the 56-hour sampling period. Each time point represents the mean ± standard error. These profiles showed significant daily variation (*P* < 0.001). The calculated area under the curve did not differ between the first and second night (*P* = 0.54).

During the 56-hour sampling period, all participants showed an expected diurnal variation in plasma melatonin (*P* < 0.001, Friedman test) (Figure 1) and cortisol (*P* < 0.001, F = 6.7, df = 70, ANOVA) (Figure 2). Significant diurnal variation was found also under baseline (*P* = 0.002 for melatonin; *P* < 0.001, F = 5.2, df = 30 for cortisol) and sleep-deprived conditions (*P* = 0.001 for melatonin; *P* < 0.001, F = 11.6, df = 30 for cortisol).

Plasma levels of melatonin were low (<5 pg/mL) between 11:00 and 19:00 hours on both days. The peak level of melatonin reached 30.5 ± 6.1 pg/mL on the first night, 20.7 ± 5.9 pg/mL on the second night, and 28.0 ± 5.9 pg/mL on the third night at 3:00 hours. Plasma levels of cortisol were low during both nights and peaked at 393.8 ± 24.7 nmol/L on the first morning, 349.7 ± 19.7 nmol/L on the second morning, and 343.2 ± 53.7 nmol/L on the third morning at 7:00 hours. Data indicate that all participants were normally entrained to the light-dark cycle. No phase shift in melatonin or cortisol peak was noted during the periods.

The area under the curve for melatonin was 188 ± 40 pg/mL on the first night and 126 ± 31 pg/mL on the second night (*P* = 0.03, Wilcoxon test). The area under the curve for cortisol was 1583 ± 199 nmol/L on the first night and 1441 ± 100 nmol/L on the second night (*P* = 0.56, Wilcoxon test).

### Daily variation of clock genes in PBMCs.

ANOVA revealed a significant diurnal variation in the expression levels of *hPer2* and *hBmal1* under baseline (*P* < 0.001, F = 19.7, df = 30 for *hPer2* and *P* < 0.001, F = 17.6, df = 30 for *hBmal1*) and sleep-deprived conditions (*P* < 0.001, F = 9.2, df = 30 for *hPer2* and *P* < 0.001, F = 13.2, df = 30 for *hBmal1*). The mRNA levels of both genes were elevated during daytime activity and low during the night.

**Figure 3:**
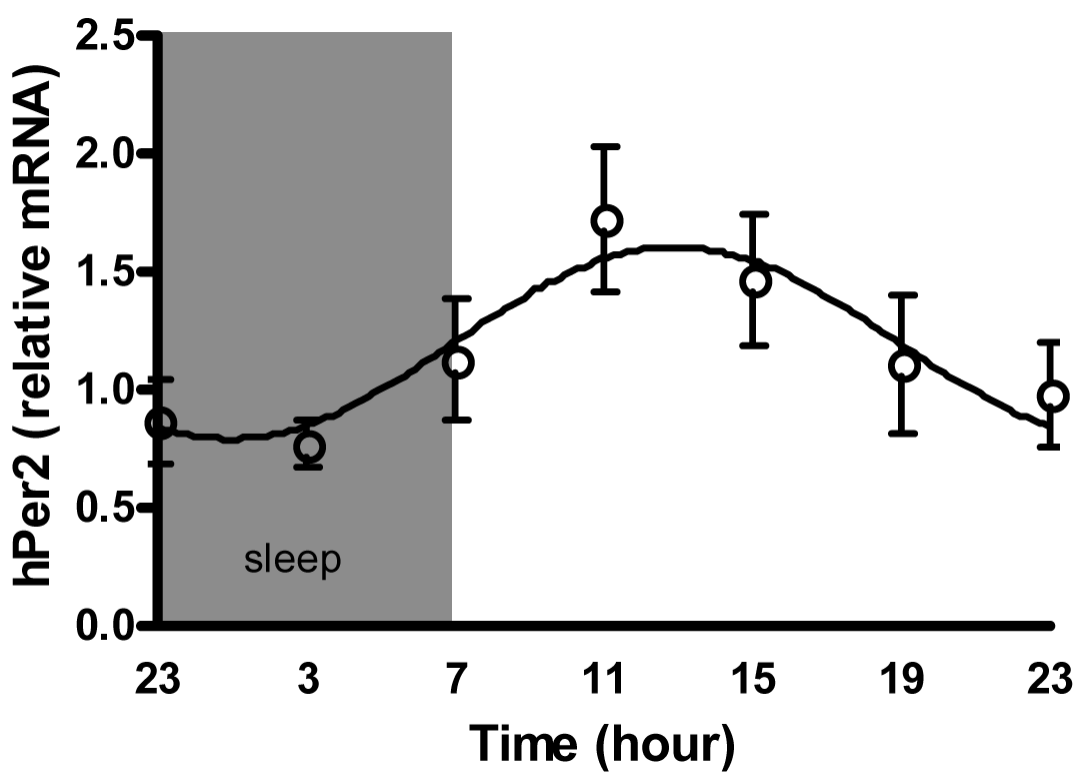
mRNA levels of the *hPer2* gene expressed as a relative ratio to the internal control gene *36B4* in human peripheral blood mononuclear cells during baseline conditions. Each time point represents the mean ± standard error. The best-fit 24-hour single cosine curve is shown (R^2^ = 0.22).

**Figure 4:**
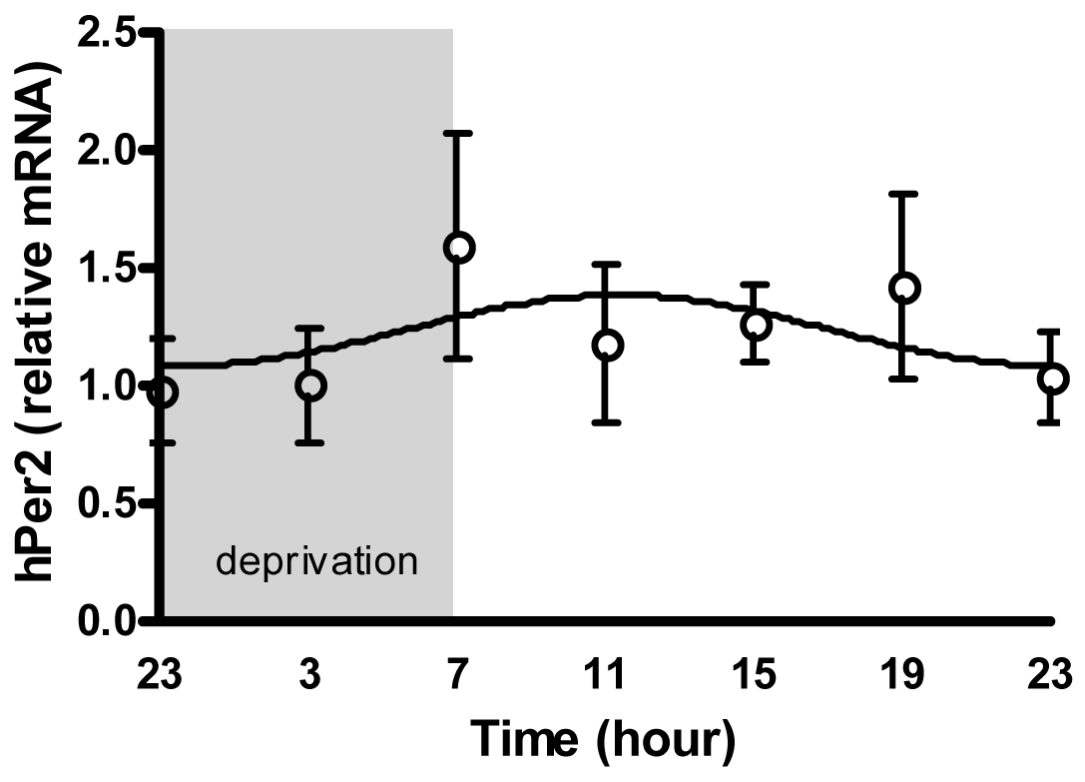
mRNA levels of the *hPer2* gene expressed as a relative ratio to the internal control gene *36B4* in human peripheral blood mononuclear cells during sleep-deprived conditions. Each time point represents the mean ± standard error. Profiles did not show significant circadian rhythm (R^2^ = 0.02).

**Figure 5:**
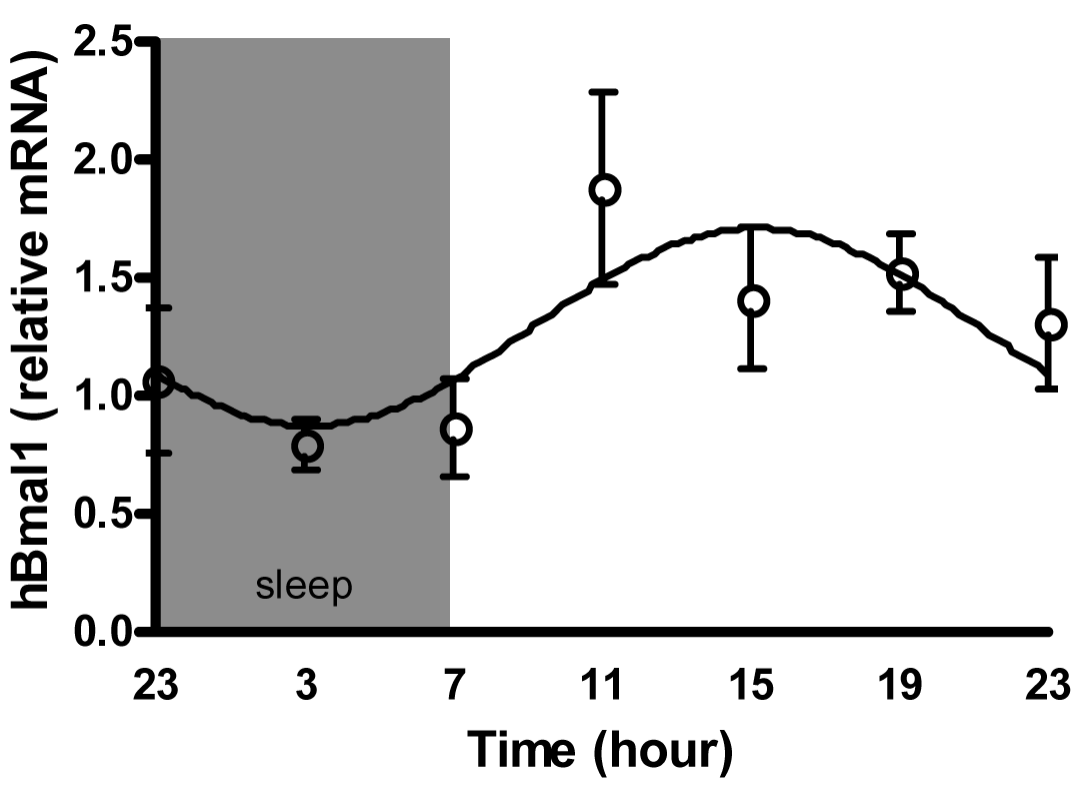
mRNA levels of the *hBmal1* gene expressed as a relative ratio to the internal control gene *36B4* in human peripheral blood mononuclear cells during baseline conditions. Each time point represents the mean ± standard error. The best-fit 24-hour single cosine curve is shown (R^2^ = 0.16).

**Figure 6:**
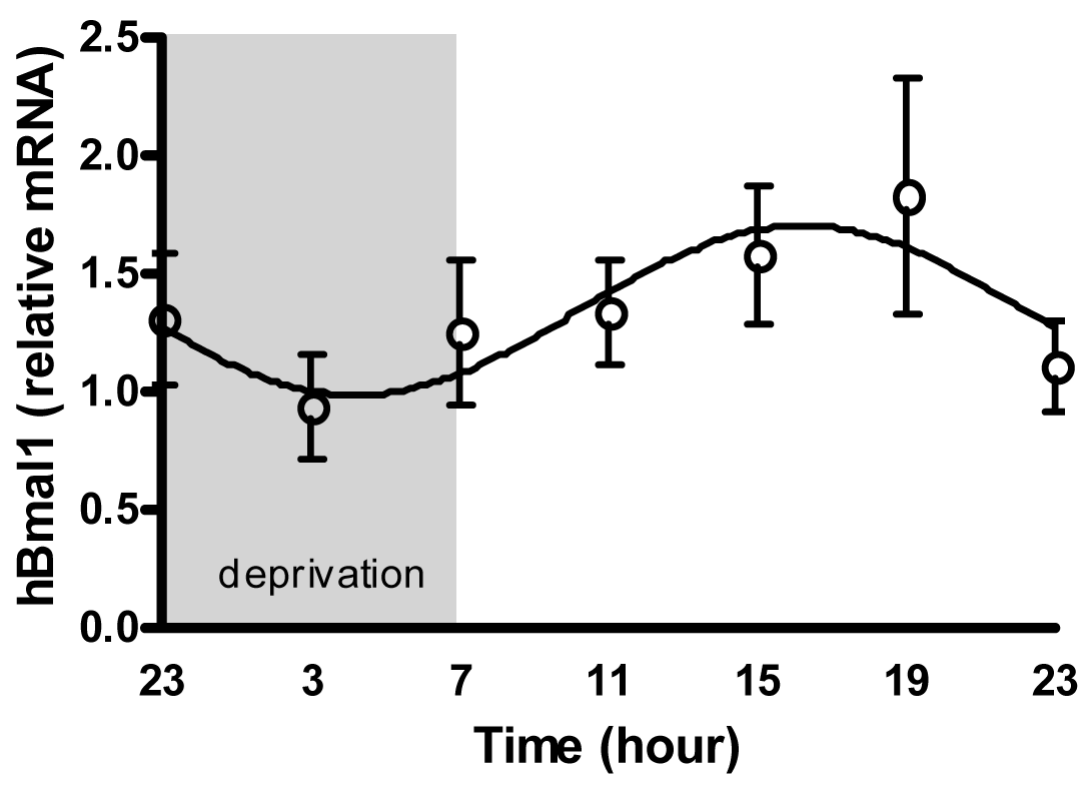
mRNA levels of the *hBmal1* gene expressed as a relative ratio to the internal control gene *36B4* in human peripheral blood mononuclear cells during sleep-deprived conditions. Each time point represents the mean ± standard error. The best-fit 24-hour single cosine curve is shown (R^2^ = 0.11).

**Figure 7:**
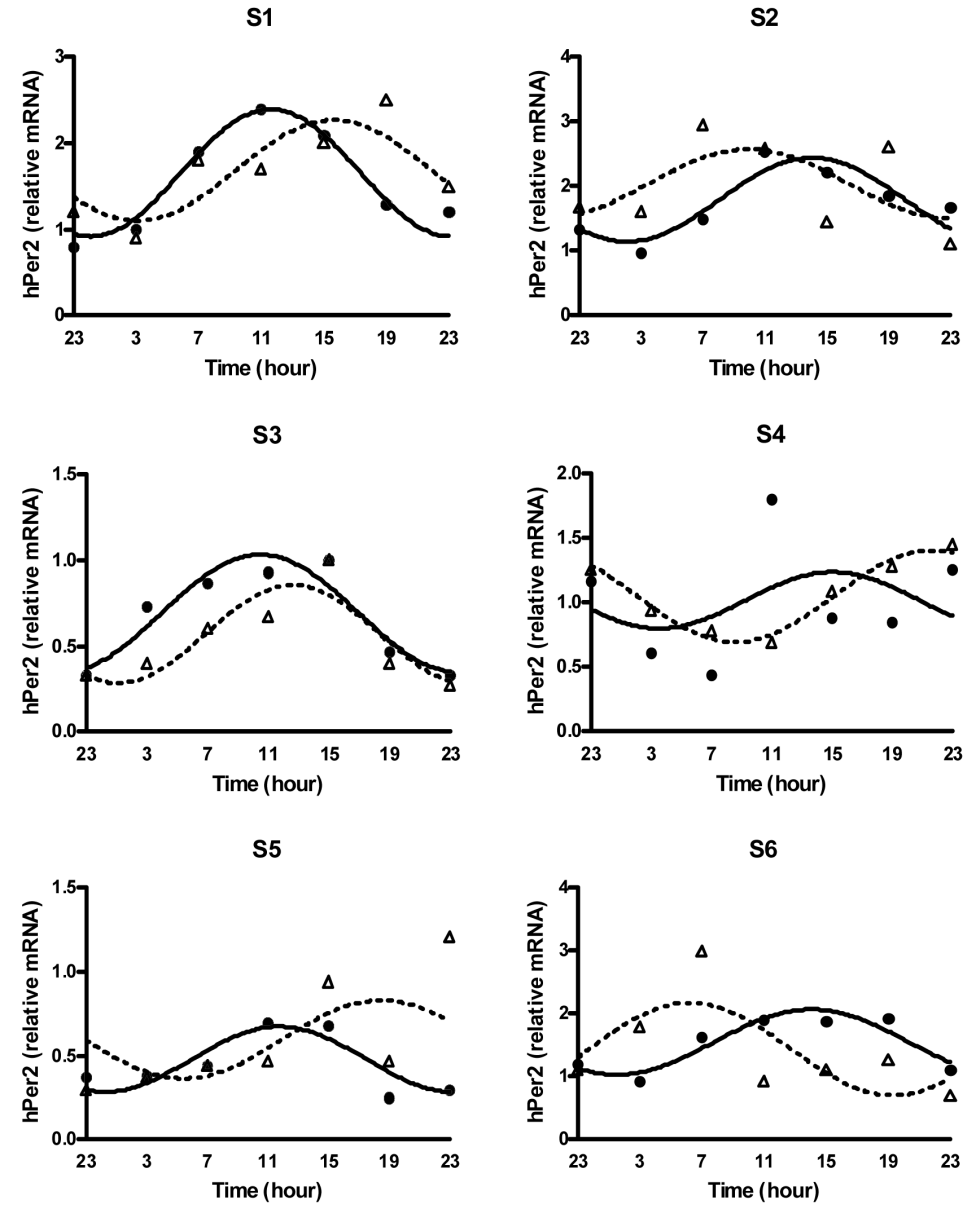
The profiles of *hPer2* mRNA expression in human peripheral blood mononuclear cells for each individual during baseline and sleep-deprived conditions. The best-fit 24-hour single cosine curve is shown. Solid line represents oscillation in baseline conditions (black dots) while dashed line represents oscillation in sleep-deprived conditions (white triangles). R^2^ = 0.94 and 0.66 for S1, R^2^ = 0.82 and 0.35 for S2, R^2^ = 0.89 and 0.77 for S3, R^2^ = 0.12 and 0.96 for S4, R^2^ = 0.76 and 0.25 for S5, and R^2^ = 0.86 and 0.49 for S6 in baseline and sleep-deprived conditions.

**Figure 8:**
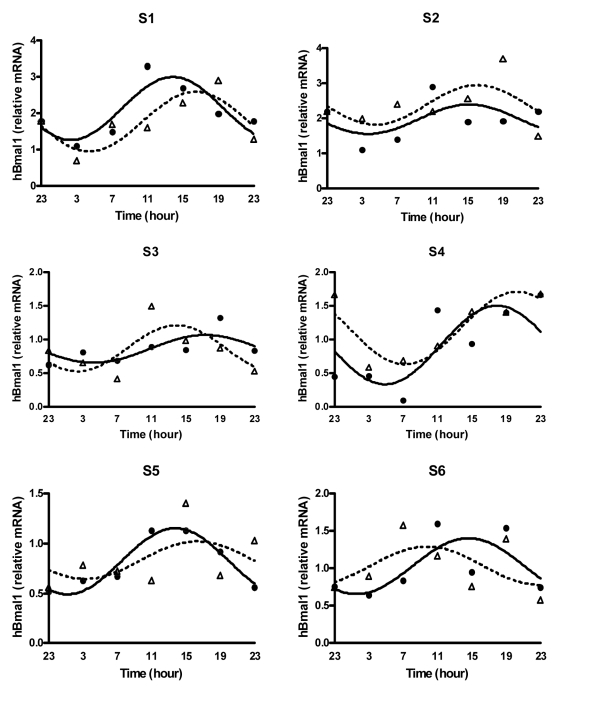
The profiles of *hBmal1* mRNA expression in human peripheral blood mononuclear cells for each individual during baseline and sleep-deprived conditions. The best-fit 24-hour single cosine curve is shown. Solid line represents oscillation in baseline conditions (black dots) and dashed line represents oscillation in sleep-deprived conditions (white triangles). R^2^ = 0.76 and 0.68 for S1, R^2^ = 0.27 and 0.34 for S2, R^2^ = 0.46 and 0.52 for S3, R^2^ = 0.51 and 0.79 for S4, R^2^ = 0.93 and 0.22 for S5, and R^2^ = 0.52 and 0.29 for S6 in baseline and sleep-deprived conditions.

Statistical analysis with the single cosinor method revealed a significant circadian variation in the expression of *hPer2* under baseline conditions, but not under sleep-deprived conditions (Figure 3 and Figure 4). The peak expression of *hPer2* under baseline conditions was at 13:55 ± 1:15 hours. It also revealed a significant circadian variation in the expression of *hBmal1* under baseline and sleep-deprived conditions (Figure 5 and Figure 6), with the peak expression at 16:08 ± 1:18 hours under baseline and at 17:13 ± 1:35 hours under sleep-deprived conditions (Table 1). No significant phase shift in *hBmal1* expression was noted during the periods. Significant circadian variation of *hPer2* expression was found in five individuals under baseline and in two individuals under sleep-deprived conditions (Figure 7). In these two individuals, the peak expression of *hPer2* expression shifted from 12:37 hours to 16:34 hours and from 11:27 hours to 13:37 hours, respectively. Significant circadian variation of *hBmal1* was found in two individuals under baseline and in only one individual under sleep-deprived conditions (Figure 8) with a phase shift from 14:52 hours to 17:27 hours (Table 2).

**Table 1:** Normalized data for the expression of *hPer2* and *hBmal1* in human peripheral blood mononuclear cells during baseline and sleep-deprived conditions*

Conditions	Variable	Mesor ± SE	Amplitude ± SE	Acrophase ± SE (hours)	ANOVA		
					F-value	*P*	df
Baseline	*hPer2/36B4^†^*	1.20 ± 0.09	0.41 ± 0.12	13:55 ± 1:15	19.7	0.001	30
	*hBmal1/36B4*	1.29 ± 0.10	0.42 ± 0.15	16:08 ± 1:18	17.6	0.001	30
Sleep-deprived	*hPer2/36B4^†^*	1.24 ± 0.15^§^	0.15 ± 0.15^§^	11:56 ± 4:35^§^	9.2	0.001	30
	*hBmal1/36B4^‡^*	1.34 ± 0.11	0.36 ± 0.17	17:13 ± 1:35	13.2	0.001	30

*Abbreviations: SE – standard error; ANOVA – analysis of variance; df – degrees of freedom.

†Expression of *hPer2* as a relative ratio of the constitutively expressed *36B4* gene.

‡Expression of *hBmal1* as a relative ratio of the constitutively expressed *36B4* gene.

§Statistically not significant oscillation.

**Table 2:** Normalized data for the expression of *hPer2* and *hBmal1* in human peripheral blood mononuclear cells for each individual during baseline and sleep-deprived conditions. Statistically significant amplitudes (ie, 95% confidence interval does not include zero) are in bold

Participant	Baseline conditions				Sleep deprived conditions			
	hPer2/36B4*		*hBmal1/36B4^†^*		hPer2/36B4*		*hBmal1/36B4^†^*	
	amplitude	acrophase (hours)	amplitude	acrophase (hours)	amplitude	acrophase (hours)	amplitude	acrophase (hours)
S1	**0.7368**	**12:37**	**0.8628**	**14:52**	**0.5830**	**16:34**	**0.8172**	**17:27**
S2	**0.6474**	**15:05**	0.4193	16:10	0.5342	10:56	0.5646	17:03
S3	**0.3419**	**11:27**	0.2051	18:16	**0.2850**	**13:37**	0.3413	14:54
S4	0.2178	16:03	0.5830	18:53	**0.3547**	**21:36**	**0.5386**	**21:12**
S5	**0.1939**	**13:14**	**0.3304**	**14:42**	0.2323	19:27	0.1878	17:16
S6	**0.5165**	**15:01**	0.3727	15:35	0.7344	6:14	0.2549	10:45

*Expression of *hPer2* as a relative ratio of the constitutively expressed *36B4* gene.

†Expression of *hBmal1* as a relative ratio of the constitutively expressed *36B4* gene.

## Discussion

The present study demonstrated the circadian expression of *hPer2* and *hBmal1* together with plasma melatonin and cortisol levels during a 56-hour protocol, which included 40-hour acute sleep deprivation under light conditions. We found a significant variation in *hPer2* and *hBmal1* expression levels under baseline and sleep-deprived conditions. Moreover, we found a significant circadian oscillation of both genes under baseline conditions and of *hBmal1* under sleep-deprived conditions. There was no reliable phase shift in the expression of *hBmal1* between baseline and sleep-deprived conditions. However, the pattern of *hPer2* showed no circadian oscillation under sleep-deprived conditions and significant circadian oscillation in only two participants. The plasma melatonin and cortisol rhythms confirmed a conventional alignment of the central circadian pacemaker to the habitual sleep/wake schedule. As expected, the peak plasma melatonin concentration occurred near the middle of the habitual sleep period, while the peak plasma cortisol concentration occurred in the early morning. The area under the curve for plasma melatonin was significantly reduced during the sleep-deprived night, which was expected due to light exposure during sleep deprivation.

*HPer2* and *hBmal1* oscillated nearly in phase in PBMCs. Interestingly, in previous human studies these two genes oscillated either nearly in phase (21,31) or with a considerable phase shift (34), and different chronotypes of clock gene expression patterns were proposed (31). However, our study and some previous studies may have too few participants. Still, the peak expression occurred during the usual time of activity and light exposure, which is consistent with prior observations of clock genes in the presence of the sleep/wake cycle (22,25,31). Although several studies have previously confirmed the oscillation of clock genes in PBMC in the absence of sleep/darkness episodes (21,29), we wanted to simulate directly the effect of “awake” night in light conditions. While *hBmal1* expression preserved circadian rhythm, the pattern of *hPer2* expression lost the rhythmicity throughout the sleep deprivation period. Comparison of individual cosinor analyses of *hPer2* expression was possible in 5 individuals (in one participant there was no rhythm during the baseline day) and interestingly showed loss of circadian rhythm in 3 participants and a phase shift in 2 participants. Our findings suggest that *hPer2* expression might be a more sensitive marker of the effect of sleep deprivation or light than *hBmal1* expression. A recent study has demonstrated a shift in *hPer1* and *hPer2* expression during simulated night shift work, while *hBmal1* expression did not follow the shifted schedule (30). There is evidence that acute light affects peripheral clock gene expression in humans. Blue light seemed to induce *hPer2* expression in oral mucosa samples of healthy adults during a 2-hour exposure (35) and reduced mean *hBmal1* expression in PBMCs of jaundiced neonates with covered eyes (36). However, the mechanism by which the SCN coordinates clock gene expression in peripheral tissues remains unclear. In our case, we cannot determine the specific contribution of the sleep deprivation or light exposure to clock gene expression.

Despite the strictly selected group of participants, we observed a considerable interindividual variability in phase of *hPer2* and *hBmal1* expression. Such variability has been often previously reported (31,37,38). In our case, *hPer2* expression was more variable under sleep-deprived conditions and has probably significantly contributed to the loss of group mean rhythmicity. Interindividual differences may arise through polymorphic variants of clock genes. In a British population sample, the polymorphisms in the *hPer1* and *hPer2* genes have been associated with morning-evening tendencies (38,39).

In conclusion, our study suggests that 40-hour acute sleep deprivation under light conditions might affect the *hPer2* expression in PBMCs and may lead to circadian rhythm disturbances. Further studies are needed to elucidate the acute effects of light and sleep deprivation on peripheral clock gene expression.
